# Kluyvera intermedia bacteremia with septic shock: A case report

**DOI:** 10.1016/j.idcr.2023.e01765

**Published:** 2023-04-11

**Authors:** Tomohiro Inoue, Yoshiro Hadano, Hitoshi Koga, Kazuhito Tamehiro, Syuichirou Sagara, Yuuji Tokuda, Syougo Urabe, Kohta Mukasa, Kouhei Onitsuka, Masahiro Higuchi, Toshiki Sudo, Takaaki Mabe, Kiyosiro Toyama, Aoi Ichikawa, Syunsuke Hisaka, Tomoka Moriya, Miki Yamamoto

**Affiliations:** a Department of Emergency Medicine, Emergency Center, St. Mary's Hospital, Japan; b Division of Infection Control and Prevention, Shimane University Hospital, Japan; c Department of Intensive Care, Emergency Center, St. Mary's Hospital, Japan; d Department of Emergency Medicine, Emergency Center, Urasoe General Hospital, Japan

**Keywords:** Kluyvera intermedia, Septic shock, Bacteremia

## Abstract

**Background:**

Kluyvera intermedia is a bacterium indigenous to humans. But Kluyvera intermedia bacteremia has been not reported thus far. We report a case of Kluyvera intermedia bacteremia with septic shock due to left obstructive pyelonephritis as a result of urolithiasis.

**Case presentation:**

A 66-year-old woman with septic shock due to left obstructive pyelonephritis was transferred to our hospital. Tazobactam/Piperacillin 4.5 g was administered every 8 h for 5 days. The patient's condition improved, and she was transferred back to the previous hospital. Kluyvera intermedia was obtained by blood cultures. The patient was successfully treated with a two-week course of antibiotics.

**Conclusions:**

We describe the first case of bacteremia with septic shock caused by Kluyvera intermedia. Kluyvera intermedia can be a causative pathogen of septic shock. Since this bacterium has not been reported in the past, we expect further reports and the accumulation of cases in the future.

## Background

Septic shock due to obstructive pyelonephritis (as a result of urolithiasis) is a relatively common disease, bacteremia of the Kluyvera species is a relatively recently discussed bacterium [1], [2], [3], [4], [5], [6] and there have been no reports on Kluyvera intermedia. In this report, we describe the first case in which Kluyvera intermedia was detected in blood cultures during the course of treatment for septic shock.

## Case presentation

A 66-year-old woman with a history of DM presented with left ureteral calculus detected by her previous physician. Relevant comorbidities included left ureteral calculus, DM, hypertension, and cerebral hemorrhage. Her previous physician scheduled ESWL for the left ureteral calculus. She developed a fever and visited her previous physician the same day. She was then referred to our hospital, a tertiary care facility, for transport due to a drop in blood pressure at the previous hospital the same day.

**Fig. 1:**
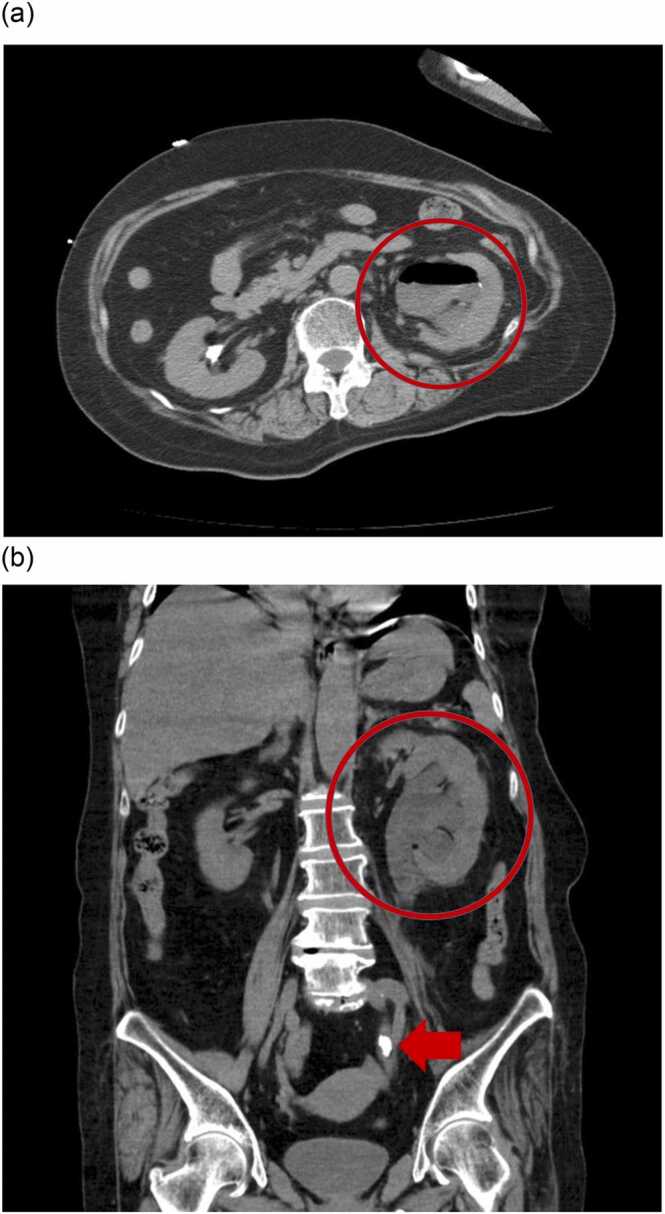
Non enhanced abdominal CT showed a stone in the left ureter and left hydronephrosis with gas images in the left renal pelvis. A) Axial view. B) Coronal view. *Note*: Circles indicate hydronephrosis; arrows indicate stone.

On physical examination upon arrival at the hospital, her blood pressure was 82/59 mmHg, pulse rate was 115 beats per minute, body temperature was 39.0 ℃, respiratory rate was 20 breaths per minute, and oxygen saturation was 95 % on room air. Her state of consciousness was maintained. qSOFA was 2 points (respiratory rate, blood pressure). Results of routine laboratory tests showed elevated leukocytes (15,220/㎕; normal range 3300–8600/㎕) with 99 % neutrophils, 1 % lymphocytes, elevated C-reactive protein (19.72 mg/dL; normal range 0.00–0.14 mg/dl), elevated glucose (292 mg/dL; normal range 73–109 mg/dL) and elevated HbA1c (11.8 %; normal range 4.9–6.0 %). Urinalysis showed elevated urinary red and white blood cell counts. Chest radiography revealed no abnormalities. Non enhanced abdominal CT showed a stone in the left ureter and left hydronephrosis with gas images in the left renal pelvis (Fig. 1).

Initial evaluation in the emergency room determined that the patient was in septic shock due to left obstructive pyelonephritis and she was admitted to the ICU. Antimicrobials were started using Tazobactam/Piperacillin (4.5 g every 8 h), empirically and two pairs of blood cultures were taken before initiating intravenous antibiotic therapy. Noradrenaline was started as a hypertensive agent and was increased to a maximum dose of 0.05γ (㎍/kg/min). On day 3, blood pressure stabilized to 110/70 mmHg, and noradrenaline was terminated. Her general condition gradually improved. We suggested ureteral stenting, but she preferred to have the procedure done by her previous physician, so we did not place the stent at our hospital. On day 5, she was transferred to her previous physician for the purpose of ESWL, which was originally scheduled at her previous hospital. On the day of transfer, two blood cultures turned positive using the BACTEC system (Becton, Dickinson and Company, Tokyo, Japan).

The species was identified as Kluyvera intermedia and its susceptibility was determined by using the colorimetric method of the Microscan WalkAway system (Beckman Coulter, Tokyo, Japan). Urinary culture turned positive for Enterobacter cloacae. Regarding drug susceptibility, Kluyvera intermedia showed resistance to cephalosporins, so Tazobactam/Piperacillin, which is also effective against Enterobacter cloacae, was continued.

After transferring hospital, she was improved after antibiotic therapy for 14 days, and on day 28, she was discharged home after rehabilitation. ESWL was performed at a later date and no recurrence was observed. No stone component detection was performed. Eventually, we diagnosed Kluyvera intermedia bacteremia with septic shock due to left obstructive pyelonephritis.

## Discussion and conclusion

This was a case of Kluyvera intermedia bacteremia with septic shock due to obstructive pyelonephritis as a result of urolithiasis. Septic shock is a frequently encountered condition in the emergency department, and pyelonephritis is also a frequent underlying condition. The patient improved with antibiotic therapy, which was continued for 14 days.

Kluyvera is a Gram-negative rod, usually found in rivers, and human contact is through drinking water and food [1]. The first description of this bacterium is from 1936, which describes the existence of a characteristic fermentative bacterium [1]. In 1953, the name Kluyvera was proposed for some of the species formerly defined as Enterobacter because of their identical genetic phenotype [2]. Later, in 1981, this bacterium was redefined as Kluyvera spp., a new genus of bacteria in the Enterobacteriaceae family [3]. In vivo, Kluyvera spp. colonize the respiratory tract, gastrointestinal tract, urinary tract, and other organs [4]. The number of β-lactamase-producing resistant strains has been reported to be increasing in terms of drug susceptibility. Kluyvera intermedia was previously called Enterobacter intermedius. In 2005, it was redefined as a genus of Kluyvera based on biological analysis. In particular, Kluyvera intermedia is reported to be very genotypically similar to Kluyvera cochleae, both being Voges-Proskauer test positive and slightly different from other Kluyvera species [2], [3], [5]. No previous literature on Kluyvera intermedia, including original papers or case reports, can be found. In 2001, the antimicrobial susceptibility of the species they identified from reported cases of Kluyvera infections was investigated [4]. Referring to general knowledge of the Kluyvera species, they are often resistant to cephalosporin antimicrobial agents [4], [6]. Within the genus Kluyvera, there have been reports on other species such as Kluyvera ascorbate [3], [6]. No information suggesting the clinical significance of the Kluyvera species can be found in past reports [3]. What is unclear about this case is that only Kluyvera intermedia was detected in the blood culture and only Enterobacter cloacae was growing in the urine culture. It is unlikely that there were technical problems with the blood culture collection or culture methods. We also considered the possibility that Kluyvera intermedia bacteremia was caused by an infection other than urinary tract infection, but other tests did not confirm any infection other than urinary tract infection. In addition to the underlying disease of DM, it is possible that Kluyvera intermedia entered the bloodstream by some route due to a weakened immune system caused by urinary tract infection, resulting in secondary bacteremia. Since Kluyvera intermedia has only been reported in a few cases in the past, future reports and studies are needed. The biochemical colorimetric method was used for this culture test. The possibility of mistaking Enterobacter for Kluyvera by this method was investigated, but no literature could be found. Our case may suggest that although this bacterium has been found to establish itself in the human body, it is necessary to recognize Kluyvera spp. as one of the causative organisms of so-called opportunistic infections.

## CRediT authorship contribution statement

Tomohiro Inoue was responsible for the coordination and writing of the clinical case. Yoshiro Hadano was responsible for the editing & review of the clinical case. All authors contributed to the writing of the final manuscript, and agree to be accountable for all aspects of the work.

## Funding

This study did not receive any specific grant from funding agencies in the public, commercial, or not-for-profit sectors.

## Ethical approval

The need for ethics approval was waived by the ethics committee of St. Mary's Hospital.

## Consent

The patient provided written informed consent for publication of this case report in print form in English.

## Competing interests

The authors declare that they have no competing interests.
